# Retrospective Study of Recurrence and Associated Factors of Type 2 Diabetes Treated at Adama General Hospital, Oromia, Ethiopia: A Comparison of Cox-PH and Shared Lognormal Frailty Models

**DOI:** 10.1155/2022/3994622

**Published:** 2022-06-27

**Authors:** Alemayehu Legesse

**Affiliations:** Department of Statistics, College of Natural and Computational Science, Madda Walabu University, Bale Robe, Ethiopia

## Abstract

**Background:**

Recovery from type 2 diabetes is frequently recurrent, as a single patient may recover from more than one over time. The goal of this study was to know the recurrent event (time to recovery) and associated factors of type 2 diabetes in Adama General Hospital, Ethiopia, by comparing shared lognormal frailty and Cox-PH models.

**Methods:**

A retrospective analysis of 302 type 2 diabetic patients (01, 2011–01, and 2016) was considered. Descriptive statistics were used to summarize the study variables. The standard Cox-proportional hazards model and a shared lognormal frailty model have been compared. The latter model with a 95% significance level was fitted, variables with *P* value < 0.05 were considered significant, and the adjusted hazard ratio has been used to measure the strength of the risk.

**Results:**

About 56.6% of the patients recovered. The average recovery time was 33.53 (standard deviation, 20.404 ) weeks. Gender (adjusted HR = 1.168, 95% CI = (0.93, 1.46), *P* < 0.05), family history (adjusted HR = 0.765, 95% CI = (0.59, 0.99), *P* < 0.05), cholesterol level (adjusted HR = 0.738, 95% CI = (0.57, 0.96), *P* < 0.05), alcohol use (adjusted HR = 0.698, 95% CI = (0.53, 0.92), *P* < 0.05), and smoking cigarette (adjusted HR = 0.674, 95% CI = (0.51, 0.89), *P* < 0.05) were statistically significant. The estimated frailty term's variance was 0.426 (*P* value=0.028). Also, the author presents a comparison study for the same data by using a model selection criterion and suggests a better model (shared lognormal frailty model).

**Conclusion:**

Finally, the median recovery time was 30 weeks. Female patients had a better chance of recovery than male patients. A shared lognormal frailty model outperformed the Cox-PH model in fitting the data and controlling event interdependence. There was risk heterogeneity among patients. Positive family history, high cholesterol level, alcohol use, and smoking have an inverse relationship with the overall likelihood of the patients' recovery time. Therefore, future improvement measures against type 2 DM recovery should take all events (for example, the first, second, and third recovery in this study) and these identified factors into account.

## 1. Introduction

Diabetes is a severe, long-term disease that occurs when the pancreas fails to produce enough insulin (a hormone that regulates blood sugar, or glucose), or when the body is unable to use the insulin that is produced. There are two types of diabetes that are commonly encountered (type 1 and type 2). Type 2 diabetes is the most common type of diabetes, accounting for 90–95 percent of all cases [1]. Globally, the twin epidemics of diabetes and obesity are escalating [2]. Obesity rates are thought to have a direct relationship with the rapid prevalence of type 2 diabetes. In the year 2000, there were approximately 300 million obese adults [3]. Type 2 diabetes is potentially reversible before permanent beta-cell failure has occurred [4]. Elevated waist circumference, elevated triglycerides, reduced high-density lipoprotein cholesterol, elevated blood pressure, and elevated fasting blood glucose were considered major indicators of type 2 diabetes [5].

Several factors, as well as the prevalence of type 2 diabetes, have been steadily rising over the last few decades. In 2014, an estimated 422 million adults worldwide had type 2 diabetes, up from 108 million in 1980. Type 2 diabetes is becoming more prevalent in Africa, and the disease's scope is expanding. Over 12 million people in Sub-Saharan Africa are expected to have type 2 diabetes mellitus, with 330,000 dying as a result of its complications [6]. Ethiopia is plagued by a slew of diseases, the majority of which are caused by transmissible infectious diseases and nutritional deficiencies. Currently, it is dealing with an increase in the number of chronic nontransmittable diseases.

The Immune Deficiency Foundation Australia (immune DFA) reported Abyssinia to be ranked 3rd among the ten top nations in Africa with 1.4 billion type 2 DM pillow slips and an expected incidence of 3.32% by the year 2012. According to WHO estimates, the number of diabetic cases in Ethiopia in 2000 was 80,000, and this figure is expected to rise to 1.8 million by 2030 [7].

There is no known cure for diabetes. The good news for type 2 DM patients is that if insulin, medication, weight loss, physical activity, and dietary changes result in normal blood glucose levels, their diabetes is well controlled, and their risk of developing diabetes is reduced. However, this does not imply that their diabetes has resolved [8, 9]. Over time, some type 2 diabetic patients may practice more than one DM recapture. This suggests that DM remission is frequently recurrent. However, most of the studies conducted previously did not take the recurrence recovery of type 2 DM into account. For example, A. N. Terefe and A. B. Gelaw [10] have conducted a study to investigate the time to recovery of adult diabetes using the Cox-proportional hazards model and stated that covariates such as types of diabetes, fasting blood sugar at baseline, sex, and age of the patients all had a significant association with the time to first recoveries. Matthew C. Riddle et al. [11] stated that the expected recovery time of type 2 DM patients and the factors associated should be investigated more in detail.

Therefore, this study was conducted to estimate the time to recurrence recovery and the associated factors of type 2 diabetes in Adama General Hospital, Ethiopia, by comparing Cox-PH and shared lognormal frailty models based on evidence obtained from the results.

## 2. Methods

### 2.1. Study Area, Study Period, Study Design, Sample Size, and Sampling Technique

A hospital-based retrospective study was conducted from September 01, 2011, to September 01, 2016, in Adama General Hospital, and 302 type 2 DM patients were included in this study using simple random sampling (simpleRS) techniques with the lottery method. The dataset has a recurrence structure as a single patient can experience at most three recoveries.

### 2.2. Response Variable

The outcome of interest is the duration of time until type 2 DM patients' recovery occurs. The author assumed that right-censored data for the patient who has only had the initial recovery, has dropped out of follow-up, has died, has been transferred to another hospital, and has not recovered at all during the study period was used as a censor.

### 2.3. Exclusion Criteria

Patients with an incomplete information sheet, under the age of 18, type 1 diabetes, or gestational diabetes were excluded from the study.

### 2.4. Data Collection, Quality, Processing, and Analysis

In addition to the hospital's staff and professionals in the field, data collectors took part in the survey. Experts reviewed and corroborated the data, which was based on a questionnaire checklist produced by the author. Data were categorized, compiled, coded, and double-checked for accuracy and completeness. SPSS version 16 was used for data entry and processing, and Chiefly *R* version 3.3.2 was used for analysis. In summarizing the data, descriptive statistics was used. Both the standard Cox-proportional hazards model (Cox-PH) and the shared lognormal frailty model were used in this study. The author fitted both models to check which of these outperform typical survival models in terms of accurately addressing the genuine research topic and efficiently estimating the standard error associated with the parameter estimate for recurring data. This was done because different literature studies suggest that likelihood cross-validation and penalized marginal log-likelihood were used as measures of accuracy of the models. The smaller value of the former and the larger value of the latter measures indicate a better fitting of the model. As a result of these insights, the author uses a shared lognormal frailty model that meets the abovementioned model accuracy criteria to solve the problem of recurring event data. Univariable shared lognormal frailty model analysis was performed to identify variables that have a significant relation with the outcome variable at a 25% level of significance, and those variables which were statistically significant were considered in the multiple covariate analysis. Finally, multiple covariate analysis was performed to estimate the adjusted hazard ratios and to estimate different values of the random effect among the events. The *p* value associated with each parameter was estimated to determine whether or not a variable is significant, and variables with a *p* value less than or equal to 0.05 are considered important variables and, thus, significant.

## 3. Results

The goal of this study is to predict the time to recovery of type 2 DM patients and related important risk variables at Adama general hospital. In this study, the author presents a retrospective analysis of 302 type 2 diabetic patients recruited at the Adama General Hospital, Ethiopia, with follow-up from September 2011 to September 2016. As a result of the recurrent recovery of type 2 DM, there have been shown zero to three recoveries during the follow-up. Thus, 700 observations were obtained.

### 3.1. Sociodemographic Characteristics of the Respondents

According to the findings, of the 302 patients treated in the study area, more than half (154 (51 percent)) were female and 56.6 percent of them were recovered. The mean recovery time of the respondents is 33.53 (±20.404 SD) weeks (ranging from 2 to 114 weeks). Within this time span, the patient experienced at most three recoveries. The maximum weight recorded is 98 kg. The majority of the participants (139 (46.1%)) were Oromo in ethnics, and one hundred twenty-one (40.1 percent) had completed primary education. Of the participants, 135 (44.7%) were in the age category <45 years, and about 114 (37.7%) have a family history (had a family with a diabetic) of type 2 DM. Besides, one hundred sixty-four (54.3%) resided rurally, and one hundred twenty-three (40.7%) have a complication history (Tables 1 and 2). This means that the patient has other conditions, such as eye problems or kidney problems related to diabetes.

### 3.2. Behavioral and Clinical Characteristics of the Participants

Out of the total individuals in the study, only 28.8% were alcoholics (Table 1). However, various studies, including the American Diabetes Association, suggest that people with type 2 diabetes may drink a moderate level of alcohol, to reduce the risk of alcohol, for example, alcohol may interact with certain medications prescribed to diabetes, and also to determine the amount, duration, and type of alcohol, they should follow alcohol guidelines and the advice of health professionals. For example, according to the American Diabetes Association, drinking more than two drinks of alcohol per day for males and more than one drink per day for females, do not allow. This may be due to its regular, continuing, and heavy use having the potential to increase blood sugar levels and hence increasing insulin resistance. This means that consuming alcohol can make it much more difficult for people with type 2 diabetes, which is defined as having high blood sugar levels, to control their blood sugar levels. Different studies classified alcohol consumption as light (0–12 g/d), moderate (>12–24 g/d), or heavy (≥24 g/d) [12, 13]. Similarly, the study done in China was defined risk levels as follows: high-risk drinkers (>60 g/d for men, >40 g/d for women); medium-risk drinkers (>25 to 60 g/d for men, >15 to 40 g/d for women), low-risk (>0 to 25 g/d for men,>0 to 15 g/d for women), and nondrinkers [14].

Similarly, 21.5 percent of patients were smokers (Table 1). As various study results indicate, smokers are more likely to get an increased risk of type 2 diabetes. In fact, their chance of diabetes goes up by thirty percent to forty percent when compared to those who do not smoke. In the patients, this leads to complex blood sugar level control. Besides, it can also worsen other health problems [15, 16]. This may be due to insulin resistance and exhibit several aspects of the insulin resistance syndrome of smokers [17]. The risk increases depending on the number, duration, and type of cigarettes that a diabetic person smokes [18–21]. For example, Will et al. [14] considered that people who smoke greater than or equal to one pack per day as heavy smokers and showed an increased risk of type 2 DM.

Excessive cholesterol levels have been seen in 43.4 percent of patients (Table 1). According to various sources, people with type 2 diabetes may have high cholesterol levels because their body does not use sugar properly. This can lead to high blood glucose levels and other complications, including high cholesterol. In terms of blood pressure, 47 percent, 35.1 percent, and 17.9 percent of the total patients were classified as normal, high, and unmanageable, respectively. Blood pressure readings of less than 120/80 mmHg, between 120/80–179/109 mmHg, and above 180/110 mmHg have been considered normal, high, and uncontrollable; the first numbers (120, 179, and 180) represent systolic blood pressure, and the second numbers (80, 190, and 110) represent diastolic blood pressure. Other diabetes-related disorders, such as kidney disease and retinopathy, are more likely to occur when the two conditions (type 2 diabetes and high blood pressure) are combined. Moreover, the median fasting blood sugar level of the participants is 199 ± 7.087 standardD) (Table 2). People with diabetes (typically type 2) have low blood glucose levels because their bodies do not use insulin properly and their blood sugar levels are higher compared to people without diabetes.

### 3.3. Model Comparison

Model comparison was done through penalized marginal log-likelihood and likelihood cross-validation (likelihood CV). The smallest likelihood CV and largest value of penalized marginal log-likelihood reveal the best model. From Table 3, the likelihood CV of the shared lognormal frailty model is lower (2.6031) when compared with those of the standard Cox-PH model (2.7946). Also, the penalized marginal log-likelihood is larger for the shared lognormal frailty model (−1919.34) relative to that of the Cox-PH model (−1923.95). This evidence indicated that in this study, the shared lognormal frailty model showed a better capability in examining interdependence within subjects, which arises from the tendency that some individuals are more prone to developing recurrent events(recovery) than others due to some hidden factors, and by adding frailty terms that follow specific frailty distribution (Table 3).

### 3.4. Risk Factors Associated with Type 2 DM Patients' Recovery

From the multiple covariate shared lognormal frailty analysis, sex (adjusted HR = 1.168, 95 percent CI = (0.93, 1.46), *P*=0.018 < 0.05), family history (adjusted HR = 0.765, 95 percent CI = (0.59, 0.99), *P*=0.041 < 0.05), cholesterol level (adjusted HR = 0.738, 95 percent CI = (0.57, 0.96), *P*=0.024 < 0.05), alcohol use (adjusted HR = 0.6976, 95 percent CI = (0.53, 0.92), *P*=0.010), and smoking habits (adjusted HR = 0.674, 95 percent CI = (0.51, 0.89), *P*=0.006 < 0.05) had a significant association with the recovery time of type 2 DM patients at 5 percent level of significance (Table 4).

## 4. Discussion

Based on retrospective data obtained from Adama General Hospital, this study attempts to estimate the time to recovery of type 2 diabetes and the major risk factors associated with it. The appropriate model (shared lognormal frailty model) for recurrent event analysis was selected based on comparison in terms of penalized marginal log-likelihood and likelihood cross-validation (likelihood CV).

According to the findings of this model, the patient's sex, family history, cholesterol level, alcohol use, and smoking habits were prognostic predictive factors in the recovery of type 2 diabetes. As a result, the study's findings are discussed as follows.

The frailty term's variance (*σ*^2^) was estimated to be 0.426 (*P* value=0.028), which was significantly greater than zero. It demonstrates that each cluster of type 2 diabetes has different values of random effects and that there is risk heterogeneity among patients. The result of model-based robust standard errors accounts for the interdependence of recurring events from the same patient.

Controlling for all other variables (including frailty), a female patient had a 1.1676 times higher risk of recovery than a male (adjusted HR = 1.1676, 95 percent CI = (0.93, 1.46)). This demonstrates that the recovery risk of female patients was increased by approximately 16.8 percent. That is, female patients had higher recovery rates and are less vulnerable to the disease than male patients. This finding is supported by study findings [3, 22], which show that female patients recover faster than males.

Similarly, after controlling for other covariates such as frailty, a patient with high cholesterol had a hazard of about 0.74 times that of a patient with normal cholesterol (adjusted HR = 0.738), 95 percent CI = (0.57, 0.96). This means that a patient with high cholesterol had a 26% lower chance of recovery than type 2 diabetes patients with normal cholesterol levels.

When other covariates and random effects were considered, alcohol users had a hazard of 0.6976 times that they did not use it (adjusted HR = 0.6976, 95 percent CI = (0.53, 0.92)). This means that the risk of recovery for alcoholic patients has been reduced by 30.24 percent. This could be due to the fact that alcohol consumption, due to its high-calorie content, can lead to excessive weight gain. Furthermore, smoking habits were significant predictors of the time to recovery in type 2 diabetes patients. When compared to nonsmokers, patients who were addicted to smoking had a lower risk (adjusted HR = 0.6735, 95 percent CI = (0.51, 0.89)). This result suggests that nonsmoking patients with type 2 diabetes have a higher chance of recovery. This could have happened because smoking raises the risk of heart attacks and strokes in diabetes. This finding is consistent with studies [2], indicating a significant negative association between smoking tobacco and type 2 DM patients.

In other words, type 2 DM patients with a positive family history, alcohol use, smoking, and a high cholesterol level had an estimated parameter ($\overset{⌢}{\beta }$) negative. This indicates that the patients' recovery risk decreased during the study period.

## 5. Conclusion

Finally, the median recovery time was 30 (20.404 SD) weeks. Female patients performed better than male patients in terms of recovery. A shared lognormal frailty model was better for fitting the data and adjusting within subject interdependence compared to the cox-PH model. There was risk heterogeneity among patients. Sex, family history, cholesterol level, alcohol use, and smoking habits all had a significant relationship with the outcome variables. Risky behaviors, such as drinking or smoking cigarettes, as well as risky clinical factors, such as high cholesterol or a positive family history, are inversely related to the overall likelihood of the patients' recovery time. Therefore, future improvement measures against type 2 DM recovery should take all events (for example, the first, second, and third recovery in this study) and these identified factors into account.

### 5.1. Strength of the Study

Unlike most of the previous studies regarding type 2 DM, this study tried to consider not only the first and second but also the third recovery to control the influence of interdependence of subsequent events on the result. Besides, it was also incorporated the shared lognormal frailty model has the power to handle such interdependence and the risk variation among the patients.

### 5.2. Limitation of the Study

Because the study used secondary data (retrospective), respondents may underestimate sociodemographic factors while overestimating health-related factors. It is preferable to use primary data to gain access to disease-related factors. Another limitation was that the findings were limited to a single institution, with no consideration given to other hospitals in the region. As a result, they cannot be applied to other institutions in Oromia. Therefore, when comparing and making decisions, this information should be used with the study's inherent limitations in mind.

## Figures and Tables

**Table 1:** Summary statistics for categorical covariates included in the study.

Covariates	Category	Status of type 2 diabetes		Total
		Censored	Event
Sex of patient	Male	65 (21.5%)	83 (27.5%)	148 (49%)
	Female	66 (21.9%)	88 (29.1%)	154 (51%)

Age	≤30 years	37 (12.3%)	66 (17.2%)	103 (34.1%)
	<45 years	62 (20.5%)	73 (24.2%)	135 (44.7%)
	≥45 years	32 (10.6%)	32 (10.6%)	64 (21.2%)

Family history	Yes	60 (19.9%)	54 (17.8%)	114 (37.7%)
	No	71 (23.5%)	117 (38.7)	188 (62.3%)

Ethnic group	Amhara	24 (7.9%)	35 (11.6%)	59 (19.5%)
	Oromo	57 (19%)	82 (27.1%)	139 (46.1%)
	SNNP	36 (11.9%)	49 (16.2%)	85 (28.1%)
	Others	14 (4.6%)	5 (1.7%)	19 (6.3%)

Education level of the patient	No education	15 (5%)	23 (7.6%)	38 (12.6%)
	Primary education	35 (11.6)	45 (14.9%)	80 (26.5%)
	Secondary education	54 (17.9%)	67 (22.2%)	121 (40.1%)
	Above secondary	27 (8.9%)	36 (11.9%)	63 (20.8%)

Place of residence	Rural	67 (22.2%)	97 (32.1%)	164 (54.3%)
	Urban	64 (21.2%)	74 (24.5%)	138 (45.7%)

Cholesterol level	Normal	67 (22.2%)	104 (34.4%)	171 (56.6%)
	High	64 (21.2%)	67 (22.2%)	131 (43.4%)

Complication history	Yes	54 (17.9%)	69 (22.8%)	123 (40.7%)
	No	77 (25.5%)	102 (33.8%)	179 (59.3%)

Alcohol use	Yes	45 (14.9%)	39 (12.9%)	85 (28.1%)
	No	86 (28.5%)	132 (43.7%)	218 (72.2%)

Initial blood pressure of type 2 DM patients	Normal	57 (18.9%)	85 (28.1%)	142 (47%)
	High	50 (16.6%)	56 (18.5%)	106 (35.1%)
	Uncontrollable	24 (7.9%)	30 (10%)	54 (17.9%)

Smoking habit	Smoker	40 (13.2%)	25 (8.3%)	65 (21.5%)
	Nonsmoker	91 (30.2%)	146 (48.3%)	237 (78.5%)

**Table 2:** Descriptive statistics for continuous covariate included in the study.

Covariate	Minimum	Maximum	Mean	Median	SD
Weight of the patients in kg	41	98	56.42	59	10.691
Fasting blood sugar in mg/dl	128	567	253.96	199	7.087
Time to recovery of type 2 DM patients in week	2	114	33.53	30	20.404

**Table 3:** Comparison of Cox-PH and shared lognormal frailty models.

Models	Standard error	Penalized marginal log-likelihood	Likelihood CV
Cox-PH	—	−1923.95	2.7946
Shared lognormal frailty model	0.0296	−1919.34	2.6031

**Table 4:** Multiple covariate analysis of the shared lognormal frailty model.

Covariate	Category	$\overset{⌢}{\beta }$	Adjusted HR	SE coef. (H)	SE coef. (HIH)	Wald	*P* value	95% CI
Sex of the patient (male = *rf*)	Female	0.1549	1.1676	0.1156	0.1156	1.3399	0.0180	0.93, 1.46
Family history (no = *rf*)	Yes	−0.2677	0.7651	0.1308	0.1308	−2.0466	0.0406	0.59, 0.99
Cholesterol level (normal = *rf*)	High	−0.3029	0.7387	0.1341	0.1341	−2.2580	0.0239	0.57, 0.96
Alcohol use (no = *rf*)	Yes	−0.3600	0.6976	0.1392	0.1392	−2.5866	0.0096	0.53, 0.92
Smoking status (no = *rf*)	Yes	−0.3953	0.6735	0.1444	0.1444	−2.7368	0.0062	0.51, 0.89

Frailty parameter, sigma square = 0.426584, SE (H) = 0.0296, P_value = 0.003								
*Rf* = reference penalized marginal log-likelihood = −1930.34								
Convergence criteria were as follows: Parameters = 5.39e−05								
Likelihood CV = the approximate likelihood cross-validation criterion in the semiparametrical case = 2.5031								

## Data Availability

The datasets supporting the conclusions of the study are included in the article, and the datasets used for analysis during the current study are available from the corresponding author upon reasonable request.

## Abbreviations

CDCP: Center for Disease Control and Prevention
CI: Confidence interval
DM: Diabetes mellitus
Fasting BS: Fasting blood sugar
Adjusted HR: Adjusted hazard ratio
Insulin DDM: Insulin-dependent diabetes mellitus
Immune DFA: Immune Deficiency Foundation Australia
kilom: Kilometer
Likelihood CV: Likelihood cross-validation
SD: Standard deviation
Significance L: Significance level
SNNP: Southern Nationality, Nation, and People
SPSS: Statistical Package for Social Science
simpleRS: Simple random sampling
Type 2 DM: Type 2 Diabetes mellitus
WHO: World Health Organization.
